# “Developing the tool SDM:KOMPASS. Supporting shared decision making implementation processes”

**DOI:** 10.1371/journal.pone.0312990

**Published:** 2024-11-18

**Authors:** Lea Lund, Dorte Gilså Hansen, Lisa Korsbek, Mette Christiansen, Karina Dahl Steffensen, Karina Olling

**Affiliations:** 1 Center for Shared Decision Making, Lillebaelt Hospital, University Hospital of Southern Denmark, Vejle, Denmark; 2 Institute of Regional Health Research, University of Southern Denmark, Odense, Denmark; 3 Mental Health Services, Vejle, Denmark; 4 University Hospital of Southern Denmark, Aabenraa, Denmark; Murcia University, Spain, SPAIN

## Abstract

Shared decision-making (SDM) involves patients in choosing their treatment or care options. SDM enhances patient engagement and treatment satisfaction. SDM has proved difficult to implement and sustain in routine clinical practice, hence a supportive tool is needed. This quality improvement study focuses on the development of a generic tool, labeled SDM:KOMPASS, which is intended to support hospital settings by facilitating the visualization of their formative progress and the setting of goals for the SDM implementation into routine clinical practice. The main objective of the present paper is to describe the development of this generic tool. A six-step development process was performed to develop a tool and investigate the tool’s overall perceived usability. Qualitative methods, such as observations, individual and focus group interviews, provided insights. A 10-item quantitative survey gauged informants’ immediate attitudes towards the tool. Purposefully sampled informants (N = 20), including healthcare professionals and patients, contributed diverse perspectives regarding; 1) The tool’s readability and clarity, 2) the construct’s domains and content, and 3) the tool’s perceived usability. In alignment with real-world challenges, SDM:KOMPASS emerges as a potentially valuable resource for healthcare organizations embedding SDM. The six-step development process revealed how the tool SDM:KOMPASS has potential to enhance SDM implementation’s manageability, goal-setting, and focus. Professionals engaged in strategic implementation within somatic and mental hospital departments find the tool potentially beneficial and feasible. The tool shows promise and usability but requires careful attention due to its comprehensiveness. The next step is to alpha test the tool in clinical practice.

## Introduction

Shared decision-making (SDM) entails a method involving patients in the selection of treatment or care options. SDM serves as a conduit to ensure enduring patient engagement and contentment with treatment outcomes. This collaborative procedure entails healthcare professionals working alongside patients to arrive at decisions regarding care, screenings, diagnostics, treatments, and follow-up, all in accordance with the patient’s expressed preferences and the best attainable information regarding available options, encompassing their disadvantages, advantages, and uncertainties [1, 2].

Although research has shown that SDM has a positive effect on a number of different parameters in health care, research also shows that SDM implies structural and cultural changes. Cultures may vary within different hospitals and departments, hence SDM has proved difficult to implement and sustain in routine clinical practice. Notwithstanding good intentions and policy statements describing better implementation of SDM into clinical routine practice, this has not occurred [1, 3–6]. We know that cultural and practical barriers partly explain this difficulty, for example shortage of accessible knowledge, skills and experience about SDM methods as well as lack of adaption to clinical systems and workflows, missed opportunities to engage patients and scarce strategies for implementation [7–10]. However, in the context of SDM implementation, there exists a gap in accessing the progress of the formative components of SDM implementation, despite available literature reporting these formative components as important for successful SDM implementation [3, 8, 11–15].

As May and colleagues states implementation cannot always adhere to a linear plan [16–18] but demands more intricate and practical guiding tools, which are currently lacking but needed.

## Proposing a practical tool

In 2019, the Region of Southern Denmark initiated efforts to create a model ensuring sustainable SDM, based on insights from large-scale real-world implementation initiatives transcending ’barriers’ and ’facilitators’. As a result, the region devised a comprehensive implementation model, SDM:HOSP [15], identifying essential components for SDM implementation.

**Fig 1:**
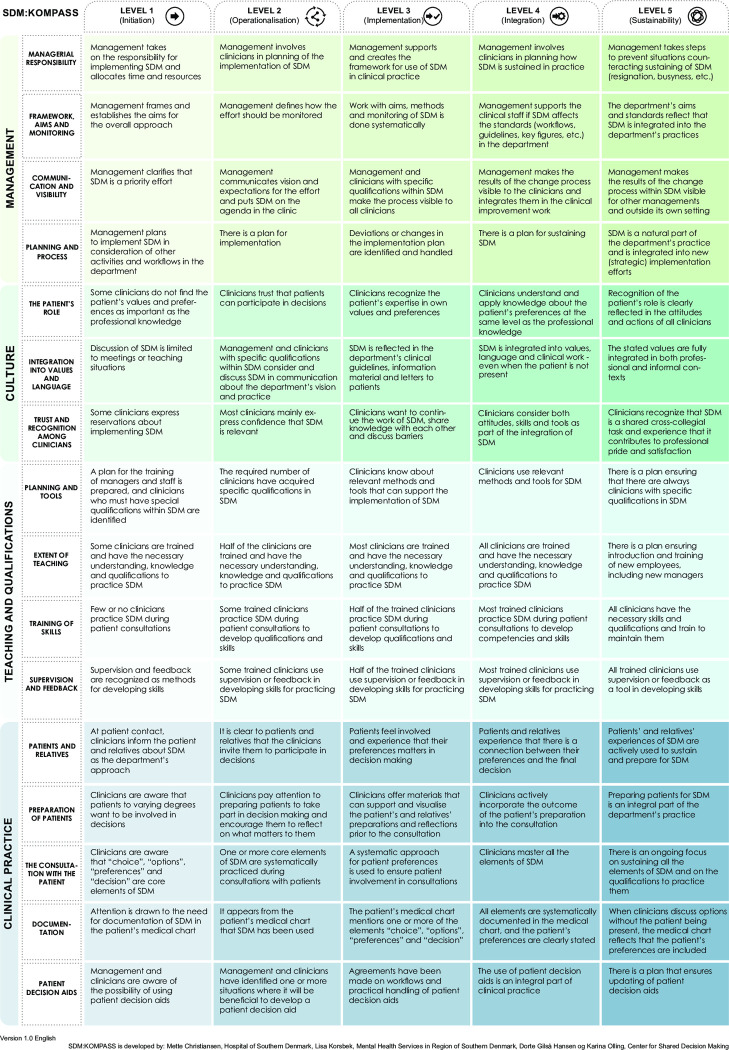
SDM:KOMPASS.

During the real-life implementation process that formed the basis of the SDM:HOSP model, it became evident that a practical, supportive tool was necessary to elucidate and visually represent the steps and different maturity levels of the implementation process. We therefore initiated a quality improvement project to create a tool that illustrated, in more detail, the necessary elements of SDM implementation. The intention from the start was to develop a tool that could be used by everyone working with SDM implementation, and not just be used narrowly for implementation following the SDM:HOSP model in Denmark, but also across borders in the field of implementation internationally. We have labelled our proposed tool SDM:KOMPASS (Fig 1).

Assessment of SDM implementation can encompass both summative and formative evaluations and while the summative approach informs about final outcomes, it may overlook the elements influencing those outcomes. Embracing a formative approach is advantageous as it assesses the process in itself, shedding light on the otherwise elusive implementation process. Moreover, in the development of other supportive tools such as Patient Decision Aids (PtDAs) within SDM implementation, transparency in the development process is pivotal [19]. This transparency remains desirable in other developmental endeavours, including the creation of SDM:KOMPASS. The label SDM:KOMPASS was chosen to illustrate the navigational purpose of the tool. Since the supportive tool was developed in a Danish setting, the spelling was decided to be a mix of the Danish wording “Kompas” and the English “Compass”.

## Aim

The main objective of the present paper is to describe the development of the generic tool for supporting the process of systematic implementation of SDM into routine clinical practice at hospital departments.

## Methods

**Fig 2:**
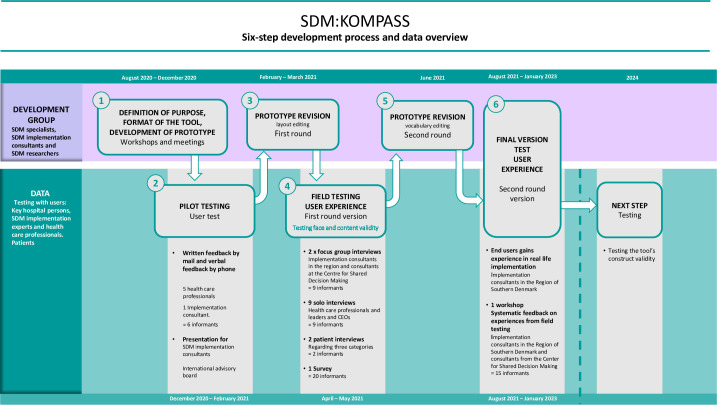
The SDM:KOMPASS six-step development process.

The development of SDM:KOMPASS as a supportive SDM implementation tool, took outset in the process model (Fig 2) lasting from August 2020 –January 2023. It consisted of a six-step development process which consisted of a triangulation of different data methods. Fig 2 provides an overview of the steps of development (illustrated in the first purple/top row) and a field testing phase collecting data on the experiences from health care professionals (illustrated in the second green/bottom row). We consider the Standards for QUality Improvement Reporting Excellence (SQUIRE guideline 2.0) [20] as the appropriate guideline to follow in reporting this paper (S1 Checklist).

### Ethical considerations

The study was conducted ensuring the data protecting guidelines. The processing of personal data is notified to and approved by the Region of Southern Denmark and listed in the internal record given the case number 24/511. The project has been approved by the Regional Committees for Health Research Ethics for Southern Denmark and has been given the case number 20232000–16. All informants gave written consent to the use of data to further develop SDM:KOMPASS and publish the process to ensure transparency. The qualitative results were anonymized by giving participants the connotation # and number. The project has not received any external funding. However, all project participants are employed by the Region of Southern Denmark and have carried out the work as part of their duties.

## Context and theoretical framework

Denmark is a relatively small country (5.6 mio. citizens) characterized by a primarily tax financed health care system. All hospital services are free. The hospital-based health care is organized in five regions making room for regional adjustment of services within the financial and national regulatory framework. Although Denmark has a long legal tradition of ensuring patient’s rights, SDM is still in an early stage [2]. But one of five regions, The Region of Southern Denmark, has been in the forefront regarding systematic implementation of SDM across clinical areas and departments [15]. In the Region of Southern Denmark, there is also continuously a focus on improvement work based on W. Edward Deming’s “System of Profound Knowledge” [21] and methods from “The South Danish Improvement Model”. The process of designing the tool itself was highly inspired of practical tools used in the South Danish Improvement model [21] and the Checklist for SDM [22] supported by the ‘Shared Decision Making summary guide’ by NHS England and NHS Improvement [23]. Moreover, real-life experience within the development group and existing theory of SDM implementation guided the development work [7, 8, 14, 16–18].

To ensure the right competencies for the development of the tool, a group consisting of SDM implementation consultants (LK, MC), SDM specialists (LK, KO) and SDM researchers (LK, DGH) from hospitals across the Region of Southern Denmark and specialists from the Center for Shared Decision Making in Denmark was established. One of the group members also had extensive experience with the South Danish Improvement model (MC).

### Informants

The informants participating (listed in Fig 2 in the green row) in this project consist of patients and health care professionals who represented both mental and somatic health care, clinical nurses, physicians and SDM implementation consultants, with different levels of experience with SDM and SDM leadership (Table 1).

**Table 1:** Overview of informants.

#	Occupation	Female / Male	Setting / location /Region	Data sources	Period
1	SDM-implementation consultant	F	Center for Shared Decision Making	FG interview + Survey	April 2021
2	SDM-implementation consultant	F	Center for Shared Decision Making	FG interview + Survey	April 2021
3	SDM-implementation consultant	F	Center for Shared Decision Making	FG interview + Survey	April 2021
4	SDM-implementation consultant	M	Center for Shared Decision Making	FG interview + Survey	April 2021
5	Communication specialist	F	Center for Shared Decision Making	FG interview + Survey	April 2021
6	SDM-implementation consultant	F	Region of Southern Denmark	FG interview + Survey	April 2021
7	SDM-implementation consultant	F	Region of Southern Denmark	FG interview + Survey	April 2021
8	SDM-implementation consultant	F	Region of Southern Denmark	FG interview + Survey	April 2021
9	SDM-implementation consultant	F	Region of Southern Denmark	FG interview + Survey	April 2021
10	Patient	F	Region of Southern Denmark	Individual interview	April 2021
11	Patient	M	Region of Southern Denmark	Individual interview	April 2021
12	Head nurse	F	Region of Southern Denmark	Individual interview + Survey	April 2021
13	Nurse specialist	F	Region of Southern Denmark	Individual interview + Survey	April 2021
14	Head nurse	F	Region of Southern Denmark	Individual interview + Survey	April 2021
15	Chief physician	M	Region of Southern Denmark	Individual interview + Survey	April 2021
16	Quality coordinator	F	Region of Southern Denmark	Individual interview + Survey	April 2021
17	Chief physician	F	Center for Shared Decision Making	Individual interview + Survey	April 2021
18	CEO	M	Region of Southern Denmark	Individual interview + Survey	May 2021
19	CMO	M	Central Region of Denmark	Individual interview + Survey	June 2021
20	CMO	M	Region of Southern Denmark	Individual interview + Survey	June 2021
21	Head nurse	F	Region of Southern Denmark	Survey	April 2021
22	SDM-implementation consultant	F	Center for Shared Decision Making	Survey	April 2021

### The SDM:KOMPASS six-step development process

We field tested the tool’s face validity in line with the description mentioned in the article on The Maturity Matrix™ [24]. When investigating the tool’s face validity we searched to get insights into how the tool ‘‘appears” to the end users, which essentially is a subjective judgement. Face validity was evaluated by inviting informant experts to review the consecutively levels within the tool and assess its suitability as a tool for appraising the SDM constructs. If the experts concurred that the levels accurately captured the intended constructs then it was assessed that the tool demonstrated face validity. Likewise we field tested the tool’s content validity [25–28]. Evaluating content validity involved a similar peer judgement performed by end users as to determine whether the tool adequately covers the essential or relevant ‘‘content” or ‘‘domains” within the concept (here it is SDM). In Step 4, data were gathered through semi-structured individual and focus group interviews, as well as surveys, in order to assess the tool’s face and content validity. A total of 20 informants provided input for this evaluation. Below we describe each individual step of the development process, including our methods of data collection.

### Step 1. Definition of purpose, format of the tool and development of prototype

The prototype was developed in the development group and made ready for pilot testing through an iterative process during six workshops and several drafts. During step 1 of the process, the development group defined the specific purpose of the tool and created various layout formats. The group conducted an initial brainstorming session drawing upon specific tools utilized for improvement initiatives [21, 29] which were well-known to the healthcare professionals in the Region of Southern Denmark. Once the group was satisfied with the prototype, it was decided to conduct a small-scale pilot test to ensure understanding among different health professionals in step 2 (Fig 2).

### Step 2. Pilot-testing

First, the pilottest included a user test and secondly, a presentation of the prototype to the SDM implementation specialists from the Region of Southern Denmark and thirdly, an online panel meeting with 16 international SDM experts (Fig 2). Six users with different expertise in SDM and SDM implementation, but all defined as being end users of the tool, were individually asked to take part in the pilot test. We drafted a preliminary guide to enhance understanding of the tool. In January 2021, the development group sent the drafts of the guide and the tool per mail to the six informants of the pilot test. They were asked to comment per mail or orally by phone. The overall question was if the idea and draft seemed useful to them, and to give input to a name for the tool. The prototype was also presented to the group of SDM implementation specialists in the Region of Southern Denmark, to assess whether the idea and scope were deemed usable in their implementation work. Utilizing ongoing illustrations of the Danish language prototype, the international Advisory Board of the Center for Shared Decision Making were engaged in an online panel meeting. During this meeting, the board deliberated upon the conceptual idea of the tool, its scope, perspectives, and potential challenges.

### Step 3. Prototype revision, first round

After the pilot test, the tool were adjusted according to the comments and suggestions through six rounds of meetings. At the sixth meeting, the methods of field test were defined, the tasks were distributed among the group members and necessary materials were developed before initiating the field test.

### Step 4. Field testing

The field-testing aimed at testing both the guide for the tool and the tool’s face validity of the layout and design, the vocabulary used for item and level headings, as well as content validity and usefulness of each level [24]. The field test focused on three topics: 1) The tool’s readability and clarity, 2) the construct’s domains and content, 3) the tool’s perceived usability in practice. We performed a qualitative study consisting of observations, individual, and focus group interviews as the main sources of insights. Additionally, we administered a ten-item survey to gain insights into informants’ immediate quantitative perceptions of the tool. The informants (N = 20) were invited using a purposive sampling strategy [30] including patient representatives (Table 1).

Table 1 provides an overview of informants participating in field testing during step 4. We sought to achieve a representative sample encompassing expertise in SDM implementation and various clinical domains. We conducted survey responses from all informants and two focus group interviews, nine individual interviews with professionals and two individual interviews with patient representatives. The informants included health care professionals from in and outside the Region of Southern Denmark.

#### Interviews–step 4 –individual and focus groups including observing thinking aloud

Two semi-structured interview guides were defined, one for the individual interviews, and one for the group interviews. The individual interviews with healthcare professionals were inspired by Hak et al.’s depiction of a think-aloud approach [31]. The specific interview process looked like this (S1–S3 Files):

1. Informants read the guide to SDM:KOMPASS, (S1 Fig) and afterwards the SDM:KOMPASS (Fig 1) and thought aloud as he/she did so.
2. Informants were provided a short questionnaire to fill in.
3. Researcher asked informants about thoughts and reactions while the informant was reviewing the SDM:KOMPASS.
4. Researcher asked additional questions regarding face validity and content validity mentioned above: 1) The tool’s readability and clarity, 2) the construct’s content, and 3) the tool’s perceived usability in practice.

In addition to listening to the thinking aloud, the observation focused on how the tool and guide were handled, read, noted on and how much time was used to go through. For the majority, the individual interview took place at the informants’ office, and the individual interviews with patients took place at virtual meetings. For the focus group interview, the informants had prepared by reviewing the tool and guide and responding to the survey beforehand. One group of informants consisted of implementation consultants with the task of implementing SDM in the clinical departments at the specific regional hospital, where they were employed. The other group of implementation consultants consisted of employees from the Center for Shared Decision Making, with the task of assisting and supporting the regional implementation consultants. The focus group interviews were conducted by two persons from the development group. One of them acted as the interviewer while the other simultaneously took real-time notes to complement the audio recording. By inviting both SDM experts, healthcare professionals, healthcare leaders and patients we sought to reach saturation. During the interview process, we reached saturation, meaning that different perspectives were presented and nothing new came through. The interviews were audio recorded and lasted for 45 to 60 minutes each. Furthermore, two individuals who took part in step 2 answered the survey only, and were not interviewed. The development group decided to include patient representatives in the field testing. However, the patient representatives were only interviewed concerning the sub-categories including patients’ or relatives’ activities or outcomes and were not included in the survey, as it focused on the hospital workflow and implementation. A structured interview guide was formulated specifically for the patient interviews, and structured notes were taken during the interview (S3 File).

#### Survey–step 4

In line with Hak et al’s description of the think-aloud approach [31] all informants completed a survey containing ten items. These items encompassed aspects such as the perceived assistance of the tool in terms of planning, goal-setting, and evaluation of the process, the significance of the four themes, overall perceived usability, elements for potential removal, and open-ended sections for informants to provide input in their own words.

#### Data processing and analysis–step 4

The audio files were carefully listened through multiple times by the interviewer. Notes were taken and for each interview, selected citations were transcribed verbatim (author DGH). Additionally, the individual interviews were transcribed to secure that no details were missed during the first round of listening. Subsequently, they were subjected to secondary analysis (author LL). Based on the interview guides (S1–S3 Files), we conducted a content analysis (authors DGH, LL) focusing on informants’ experiences and meaning regarding: 1) The tool’s readability and clarity, 2) the construct’s domains and content, 3) the tool’s perceived usability in practice. Statistical analysis of the survey data was restricted to descriptive statistical methods.

### Step 5 –Revision, second round

After the field test only a few changes were made (see Results section). The tool was sent to a linguistic expert to ensure correct use of vocabulary. Finally, SDM:KOMPASS and results from the field test, were presented for the twelve persons (hospitals directors, representatives from Center for Shared Decision Making and patient representatives) in the Regional Steering Committee who are responsible for the implementation of SDM in the Region of Southern Denmark, and they encouraged further implementation.

### Step 6 –Final version test–user experience

The development group engaged in several discussions to determine the optimal approach for introducing the tool to end users. The group reached a consensus that a bottom-up strategy was most suitable. This strategy involved simply informing key individuals responsible for implementation within the ongoing process in the Region of Southern Denmark about the tool’s purpose and potential applications.

Over a span of eighteen months, end users were provided the opportunity to field test the tool across diverse settings, accumulate experiences, and establish effective workflows. This phase culminated in a workshop that involved the participating end users. During this workshop, informants shared their insights on appropriate contexts for utilizing the tool, effective methods for integrating it, successful strategies, areas that presented challenges and they were asked if they would recommend using the tool to others working with SDM implementation. Furthermore, they offered suggestions for valuable and customized supplementary materials.

## Results

Steps 1 to 3 led to the creation of the SDM:KOMPASS tool and an accompanying guide (S1 Fig), which is presented in the end of this section. Both underwent field testing in step 4. In step 5, prototype revisions were conducted, and in step 6, real-world end user testing took place (Fig 2). The results from steps 1 to 6 will be elaborated upon in the following sections.

### Step 1 and 2. Definition of purpose, format and pilot testing

Step 1 resulted in identification of four essential themes for real-world SDM implementation: I) Leadership, organization and strategy, II) Culture, III) Teaching and clinical competences, and IV) Clinical practice. The group conceptualized each theme within five levels of implementation; Initiation, Operationalization, Realization, Implementation, Integration, Sustainability. The pilot test resulted in several practical suggestions regarding the wording, and in summary, everyone found the tool to be useful and interesting, yet comprehensive.

### Step 3. Prototype revision, first round

Some of the most important adjustments were definition and naming of sub-categories within each theme, “clinical staff” were altered to “clinicians”, and an additional sub-category named “preparation of patients” were added. The final name, SDM:KOMPASS, was decided on and the layout of the tool was settled with support from a graphic designer.

### Step 4. Field-testing

#### Interviews–individual and focus group

**Fig 3:**
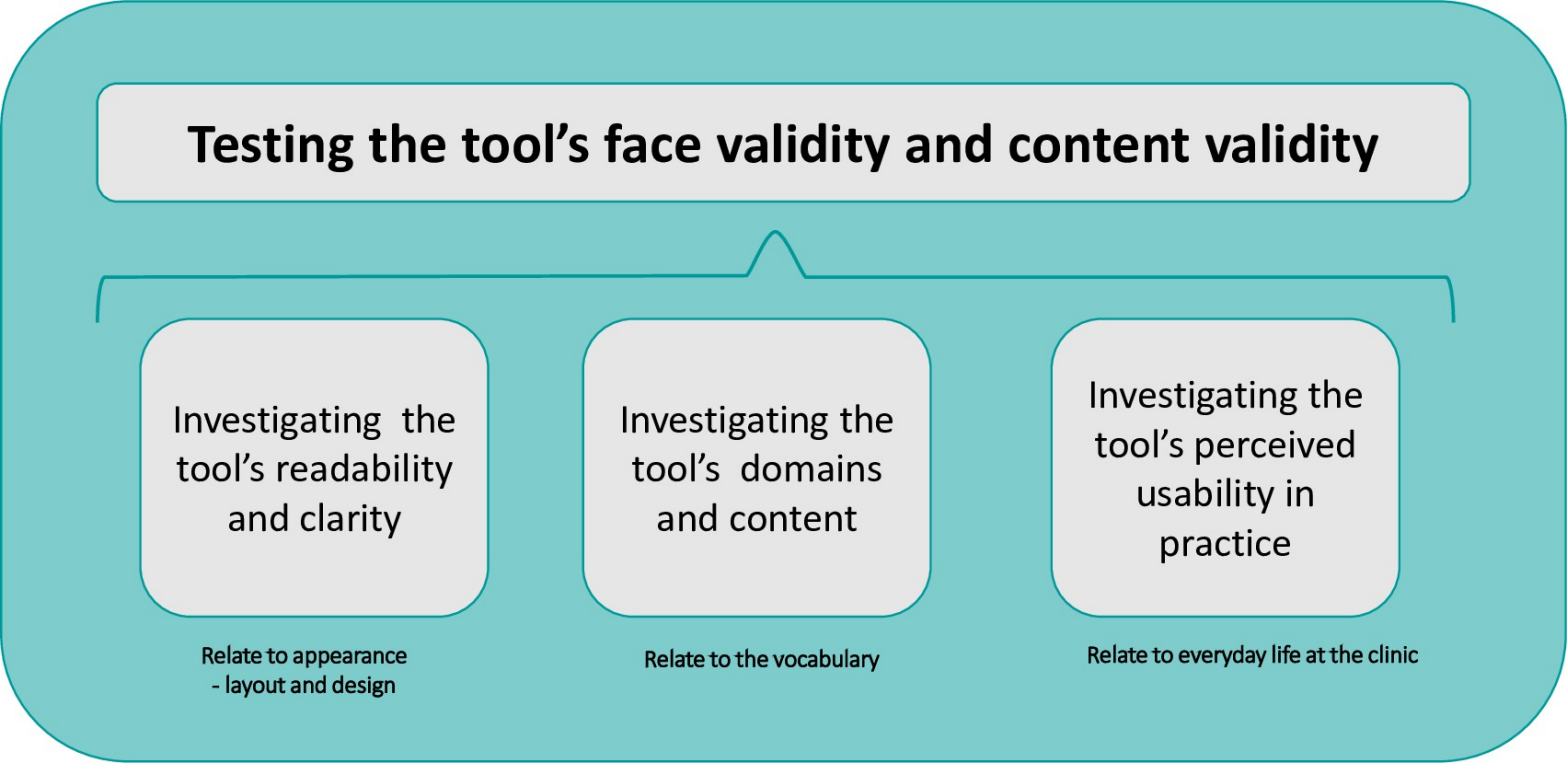
Content analysis framework–step 4.

When presenting the results derived from analysis of the interviews, including observations during the interviews and survey data (step 4, Fig 2), the three focus areas outlined in our content analysis framework (Fig 3) are utilized to affirm the positive testing outcomes of the tool concerning face validity and content validity.

#### The tool’s readability and clarity

During the development and field testing (step 2, Fig 2) and the prototype revisions (steps 3 and 5) we spent much effort in tailoring readability to the constructs in SDM vocabulary and making these operational into the clinical vocabulary used in their everyday practice. When assessing the readability and clarity of the tool, we focused on the layout and design and its visual appearance. Readability is understood as the perceived ease with which a reader can understand a written text. The readability of a text depends on its content (complexity of syntax and vocabulary) and its presentation (e.g. font size, line height, character spacing, and line length and colors), which are the typographic aspects that affect readability. The practical suggestions regarding improvement of the tool expressed during the think-aloud interviews, have been adjusted in the final version of the tool of the guide SDM:KOMPASS (S1 Fig) and the tool SDM:KOMPASS (Fig 1). Even though the informants had comments of adjustments they all found the basic elements in the layout of the tool understandable. Below we have chosen some of the most illustrative testimonials from health care professionals, managers, Chief Executive Officers (CEOs), Chief Medical Officers (CMOs) and SDM implementation consultants which show how well they experienced the tool’s readability and clarity.

A chief nurse expressed that it is “Good to have the colors that separate the four themes”(#14). And a nurse specialist stated: “You can feel that it’s a well-thought-out material. It’s nice to hold in your hands" (#13). A quality coordinator stressed the importance of recognition from other improvement models and approaches. She stated:

*“Recognizable format*, *similar to the Virginia Mason concept that we use in the quality department*, *in improvement management*. *It is easy to use*, *I can quickly read up on how to jump into it*. *The tool is well set up and it is nice that it is in line with the setup that we know with the nice colors and I am not overwhelmed by the form*, *it’s nice to have something that takes you by the hand” (#16)*

Informants highlights that the layout, choice of colors and design were easily accessible. As implementation consultants stated, the tool is “…both highly visual and operational. And it clearly illustrates the many aspects that are important for successful implementation, it provides an overview” (#1–5). A chief physician stresses that the tool is “…delicious and well thought out. Makes it easy to say yes. Energy has been spent on ’selling it’, and the colors help it to be perceived and read as steps” (#15). The tool’s readability and clarity is summed up in this statement from a nurse specialist where she underlined her experience of usability due to the readability.

*“It makes it visible*, *so instead of it being in my head what we are going to do*, *it will be more visible here [in the form–the schematic chart]*. *It will also be a chart I could hang up*. *That I could look at and maybe keep checking off*. *I like that way*, *to check off*. *Then we are this far*. *Then I think it’s nice to have those headings*. *We are still operationalizing*. *The form also clarifies our thoughts on how to structure how to get new clinicians on board”(#13)*.

#### The tool’s domains and content

As previously noted, the development group closely adhered to the Southern Danish Improvement model (39), and a crucial aspect of this tool is the operationalizability of the content related to the development of an SDM approach. Concerning content validity, it’s imperative that the tool’s terminology remains relevant to both end users and SDM experts. The development group adjusted the wording after step 4 in the development process based on comments from the informants. The quotations below illustrate the overall perceived satisfaction with the domains and content.

A CMO from a hospital in the Region of Central Jutland described the tool’s content (the CMO called it “concrete suggestions”) having great value while the suggestions are specific:”Extremely concrete and that’s good for me. The tool tackles relatively complex issues with some very concrete suggestions. That’s taking the implementation work really seriously” (#19).

An implementation consultant from the Region of Southern Denmark expressed that they find the right content choices in vocabulary use in the tool: “It seems like the right things have been thrown into the schedule. Even though it’s a big mouthful, it’s still manageable, albeit perhaps especially as a manager"(#7–9). Likewise the implementation consultants from the Center for Shared Decision Making applauded the content and wording used as they underline the importance of a recognizable structure.

*“The tool is in line with ’competency models’ already known in the clinic*. *Recognizable structure*, *step-by-step progression from novice to expert*. *It is okay to be at level 1*. *[The terms and content] supports working with one area at a time*. *Really good tool*, *readable level by level*, *makes me wise about the process*, *but time consuming*. *The tool includes all the many things we have learned are necessary [during an implementation process]*. *Grouping into themes is visual and operational*, *and makes it clear*. *But it is more a tool for managers than for the clinician ’on the floor’*, *and managers may feel it is useful because it appeals to ’winging it’*. *And the [terms and content] will be read differently by people at different levels”(#1–5)*.

Likewise a chief nurse also reflects on the aspects of levels of maturity and how the tool’s terms can help develop a common vocabulary among professionals.

*“It could be a bit of fun for the team members to assess individually and then have a team discussion*. *Make a joint decision about where we are and how we want to work with it*. *Create a common language*, *it will make good sense and for us to get to the next level*, *we can then agree on what is needed and also on our intermediate goals”(#14)*.

As we explored the informants experience of the tool’s terms and content it became clear that they especially related this to the role of the manager, and leaders of the implementation process. A nurse specialist expresses how the tool can support her work with implementing SDM while her leaders are novices into SDM.

*“The difficult thing is that the two managers [in the department] don’t know much about it [SDM]*. *And I can go back and use this schedule [SDM:KOMPASS]*, *it will help me go back and say this is what we need [refer to the content of the themes]*. *It also makes it clear to the clinicians what their role is*. *I would definitely recommend it to others and use it myself*, *and also take it to clinical conferences*, *preferably with dates on it”(#13)*

The informants described how the division of the four themes is meaningful in their daily life at the clinic to ensure they get started with a meaningful implementation. In the beneath quotation, a chief nurse contributed to content validity:

*“It’s really operational*. *It [SDM:KOMPASS]*, *can work as a process plan I can have in my whiteboard meetings*. *It’s great that it’s divided into the four groups because then it becomes clear at the meetings*. *We often work for a long time in management before the clinicians are involved*. *This will help us get the clinicians involved sooner*. *It is very clear*. *A really nice process plan*. *I will use it*. *It will make sense to take to my colleagues*, *this is what we work with*, *this is the way we think about shared decision-making*, *and there is actually also a culture and some management and competence development”(#13)*.

In line with the above statement, a quality coordinator talked about the content value of the tool as it makes the process visible to managers: “The tool makes it visible to the management how many facets are important in implementing SDM”(#16). Also a CMO appreciated the four themes chosen to operationalize the implementation of SDM in hospitals and especially he underlined the value of the tool for managers: "The four themes are very natural and right steps" (#19). Especially in relation to the leadership theme, he says:

*“It’s very coherent*. *The management team has backed it very strongly*. *They have asked about the process*. *It fits in very well with the leadership work our departmental managers have requested*. *Who has it [who is responsible for what?]*, *and what framework we have to work under? Will there be something regional*, *or is it something we have to invent locally? [the managers in his hospital ask]“(#19)*.

The informants acknowledged the tool’s terminology, choice of phrasing, and levels of maturity, which they found consistent and familiar in relation to the structure they encounter in their daily utilization of competency models already known in the clinical setting. As observed above, the informants perceived the levels of maturity as relevant.

#### The tool’s perceived usability in practice

The informants expressed their perception of SDM:KOMPASS as a valuable tool for the purpose of implementing SDM in their day-to-day clinic operations. Specifically, they elaborated on how the tool can assist them, as managers, in orchestrating the deployment and active integration of SDM. Furthermore, they noted its potential usability in keeping staff informed about the necessary steps to foster the advancement of SDM realization. As articulated by a chief nurse, the levels of maturity serve as guiding markers for the process of SDM implementation. As this chief nurse stated the levels of maturity guide the process:

*“It’s a tool that allows you to work it into the department*. *It makes good sense*. *It is a good picture of how I can see where we are and where we need to focus*. *And I can use it as a manager*. *We are a small group and we can use it to keep an overview*, *to see where we are*. *I can just pull it out*. *Refer to what we said last time and then take it from there*. *It would be good to do [a process where we use SDM:KOMPASS] for the department and for the team*, *and take them with us [by looking at the SDM:KOMPASS] so we can see we are moving forward*. *Maybe also for the outpatient clinic where they have been working with it for several years”(#14)*

Regarding the tool’s usability, it was significant for the informants to highlight that the tool can bolster the entire management team. They stressed that the different leaders should be seen as a unit that works together operating in accordance with uniform metrics and shared objectives. As pointed out by the following chief nurse:

*“It’s really good for ensuring that you get the same thing done*, *it’s good to have it so clear*. *It’s good for me to understand the process*, *so I’m more confident and can give employees direction*. *SDM:KOMPASS will help me get shared decision-making out to my functional managers so we can see how far we’ve come*. *Such an overview [from the tool] will be really good to have*. *In the long term*, *it could also be something to have in the management team as a supportive tool*. *Perhaps you could typically ask on a weekly basis how much are documented in the patient charts? And how many have been trained and then also include that in the whiteboard meeting”(#12)*

A quality coordinator expressed how she sees it as a good process to start using the tool in the implementation team and then later on it should become part of the entire department’s way of working with it. The tool will support both objectives and ensure that it becomes visible what you are working with.

*“I would start in the management/implementation team*. *But it has to be a staff tool—where are we—where are we going? And then used in the same way as the improvement boards*. *You also need to look at the low-hanging fruit so it doesn’t all feel like really hard work*. *It could work as a motivational tool in the process showing us that we have reached/have managed*, *etc*. *It must be experienced as an opportunity to be realistic about one’s own department*. *It will be good to have at hand when we need to see how far we are*. *And I will be happy to have it myself*. *Both for shared decision-making and also in many other contexts*. *We can use it to talk about where we want to go as a goal*. *And it provides an opportunity to make visible the many things that we as leaders must need to see “the bigger picture” and be held accountable“(#16)*

The above two quotes underline how the tool clarifies the department’s progress in the process aka the maturity aspect of the tool in the formative evaluative process supporting the leadership team in succeeding in their responsibility of the process. A chief physician also stated how the leaders can use this tool as a joint venture and setting the scene for the next steps to come:

*“I want to use it together with my new manager when we have to introduce shared decision- making in the department*. *If we are going to do this [Shared Decision-Making]*, *we need resources and that the development process will take half or a whole year and this is well signaled by the chart*. *It also provides an opportunity for reflection*. *I reflect on where we are in our clinic—both the team and the department as a whole*. *It gives us the opportunity to motivate clinicians*, *we can identify focus areas*, *e.g*. *supervision and patient preparation*, *where we are ’behind’*. *It is good for highlighting areas of action*. *My immediate assessment is that it is a great tool*. *I would like to use that and take it with me to my new position*. *Really good and thorough*. *Clear and concise*. *Makes it easy to say yes and to get started and allocate resources”(#15)*

The Implementation consultants from the Region of Southern Denmark agreed that the tool has practical benefits during a focus group interview. They mention the following points as listed below:

*SDM:KOMPASS is a concrete working tool and can be used as a management tool*. *It can be used for internal feedback and for clarifying different perspectives in e.g*. *a department*. *And it can be used to identify*, *formulate and set implementation goals at different levels and stages of development*. *The tool can be used to guide action*, *including in the sustainability process*. *Furthermore it can be used during specific discussions with or in departments to find out where we are now and where we would like to go at the moment*. *It is experienced especially as a management tool and as an auditing tool*, *especially because of the goal hierarchy (#6–9).*

The implementation consultants further debated about the possibilities of uneven assessment in the same department. They underline the importance of the aspect of different assessments gives the opportunity to different voices and experiences when processing a formative evaluation. In line with the chief nurse (#14) above stating that the tool can foster a common vocabulary:

*“It is only good to have different assessments of where you are in the process*, *because it can help to clarify the different perspectives and assessments*. *Differences in assessment are therefore not a problem*, *but can lead to some good discussions*. *It is therefore also important that not only one person is evaluating the implementation effort with this tool*, *but several people*, *i.e*. *the team itself/the people who are in the process”(#6–9).*

This is in line with the implementation consultants from the Center for Shared Decision Making, who underline the tool’s role as a mediating the dialogue, as they explained:

*“The tool is a good starting point for planning; agreeing on the starting point; disagreement or uneven assessments will be a good starting point for dialog*. *In particular*, *[the themes] ‘clinical practice’ and ‘culture’ will be read differently depending on position”(#1–5).*

The implementation consultants from the Region of Southern Denmark additionally indicated that the tool holds considerable usability for managers, aligning with the previous observations within the construct’s content. However, they anticipated that for clinicians, it might initially appear somewhat overwhelming, as articulated by one of the informants:

*"…"Uh*, *that’s quite extensive*. *But once you’ve spent a bit of time on it*, *it looks really nice*, *and the structure is well-designed and covers the necessary aspects‥(…)… The document [SDM:KOMPASS] may seem overwhelming in its entirety*, *so it is important to emphasize that it can be used in “chunks” and in relation to the current focus area"(#6–9).*

Summarizing the qualitative data from step 4, it is evident that SDM:KOMPASS is regarded as a valuable tool, particularly for managers and those being in front of the implementation process.

#### Surveys

As described in the methodology section, the informants who were interviewed completed a ten item survey (Table 2) prior to their qualitative interviews. Before responding to the survey, they were presented with SDM:KOMPASS and the guide. No discussions about the tool took place prior to survey completion. Using a Likert scale of 1 to 5 (where 1 is: ‘not at all’ and 5 is: ‘to a very high degree’), a majority of all respondents have indicated agreement to a ’high degree’ or ’very high degree’ for the initial nine questions related to the face validity of the SDM:KOMPASS tool. This underlines the strength of face validity and content validity, as evidenced in the survey results.

**Table 2:** Survey results.

N = 20	Not at all	To a minor degree	To some degree	To a high degree	To a very high degree
Item 1. Can the tool support you in planning the implementation of SDM?	0	2	2	13	3
Item 2. Can the tool support you in assessing how far you have come with SDM implementation	0	0	1	12	7
Item 3. Can the tool support you in defining goals for your implementation of SDM	0	0	5	11	4
Item 4. The theme “Leadership” is relevant for SDM implementation	0	0	1	3	16
Item 5. The theme “Culture” is relevant for SDM implementation	0	0	0	3	17
Item 6. The theme “Teaching and Qualifications” is relevant for SDM implementation	0	0	0	5	15
Item 7. The theme “Clinical Practice” is relevant for SDM implementation	0	0	0	4	16
Item 8. I could use the tool in the current version if and when I am implementing SDM	0	1	3	12	4
Item 9. On a scale from 1–5 where 1 is worst and 5 is best, how useful do you think the tool would be to a team or department who is going to implement SDM	(1) 0	(2) 0	(3) 1	(4) 12	(5) 7
Item 10. Are there elements you wish to delete? If yes, please describe	Yes ⧠ No ⧠ (free text)----------------------------------------------------------				

Questionnaire item ten encompassed an open-text section, allowing informants to provide further insights into their assessments. These inputs were considered by the development group. Presented below are two quotes extracted from the open-ended responses, which encapsulate the informants’ reflections on the tool’s usability.

*“At first glance*, *the compass [SDM:KOMPASS] seems a bit large and overwhelming*. *However*, *when you sit down with it*, *it works fine*. *But it might be concerning that some clinicians give up on it*, *due to its size*. *On the other hand*, *I don’t think any elements should be removed*. *For some departments it can definitely be a useful tool if it is consistently used if the department sees value in it*, *and if it’s prioritized*. *I might be worried that in a busy everyday setting in some departments*, *it could be challenging to manage*. *Perhaps it could be integrated as a preparation for meetings with the departments*, *where discussions can be based on areas that aren’t progressing and need to be worked towards*. *It is difficult to say how useful the tool is and whether it can be used in its current form before you have sat with it in a department”.*

Another informant elaborates on this in the open-ended descriptions, emphasizing how the tool can help evaluate the maturity level of the department:

*“The tool clarifies the importance of leadership responsibility*. *It creates an overview and offers both department and management insight into what needs to be done*, *how far we are and where we would like to end up*. *I think it gives a good overview of what needs to be addressed*. *It would be good to show the department where we are in the process”.*

These responses, coupled with the overall positive survey feedback (Table 2), signal that our preparatory work was primed for real-world testing across hospital departments and units. In summary, the 20 questionnaire responses affirmed that SDM:KOMPASS effectively targets relevant themes and proved immediately beneficial for teams or departments implementing SDM. In essence, the face validity and content validity aligned with end users’ perspectives, considering these assessments are inherently subjective. The data analysis from step 4 informed minor adjustments for step 5.

#### Step 5 –Revision, second round

During this step, an additional sub-category labeled "documentation” was introduced and a few wording adjustments were made. For instance, the sub-category "relatives" was incorporated into an existing sub-category. The arrangement of sub-categories within the "Management" theme was modified based on insights from interviews, with "Managerial responsibility" placed first to emphasize its significance. Following these refinements, SDM:KOMPASS underwent linguistic review, resulting in minor refinements. The final version of the guide (S1 Fig) and the tool SDM:KOMPASS (Fig 1) was now ready to be introduced to end users.

#### Step 6 –Final version test–user experience

The commencement of the bottom-up approach for the introduction of the SDM:KOMPASS tool to the SDM implementation consultants culminated in a workshop were 15 consultants actively participated. During this workshop, each informant was tasked with delivering a presentation elucidating the extent and manner of their incorporation of the SDM:KOMPASS tool within the context of the SDM implementation process in their hospital. Additionally, the informants were required to expound upon the methodologies employed in this integration. Predominantly, it was observed that the tool had been assimilated into the educational activities directed towards leaders and health care professionals, as well as incorporated into various planning activities conducted throughout the implementation process. It is noteworthy that the modalities employed for this integration exhibited some diversity; however, the prevailing method consisted of presenting SDM:KOMPASS tool to pertinent stakeholders within the clinical departments, subsequently followed by a brief workshop for their practical engagement. Asked if anything had not worked well, one of the consultants claimed; “….it takes time to familiarize yourself with the tool. But that’s more a reflection of the complexity of the implementation than the format of the tool”. Another consultant indicated; “… It does not work just to hand out the tool at meetings or educational classes”. There were also given examples of what worked well. One consultant said:

*”….It worked well to visualize in joint discussions; it raises awareness of challenges*, *and when several teams/departments look at them together*, *they also realize that they may be the same challenges–making it a mutual sparring tool”.*

Another consultant told about a statement from fellow manager working with patient involvement. The manager said; “….this is the most concrete and useful management tool for the work with patient involvement I have ever seen”. All 15 implementation consultants indicated they would recommend using SDM:KOMPASS for future implementation work.

## Discussion

The present quality improvement study embarked on a comprehensive journey of developing, refining, and field testing the tool SDM:KOMPASS which aimed at guiding healthcare organizations in the practical adoption of SDM. Through an iterative process, the development group systematically crafted SDM:KOMPASS, and the subsequent field testing and user experiences outlined in steps 4 to 6 to offer insightful reflections on its potential impact within clinical practice.

The intention was to develop a tool which could be used across Danish borders and outside of the SDM:HOSP model. The possible alignment in the Danish implementation model SDM:HOSP with elements from other large scale SDM implementation efforts like MAGIC [7, 32] and the German SHARE TO CARE [33, 34], provides an opportunity to incorporate SDM:KOMPASS as a supportive formative element. Another practical tool like “Checklist for SDM” [22] would also be of value, and the choice between the two concrete and practical tools would depend on the preferred format and compatibility with the SDM implantation in question. The developers posit that SDM:KOMPASS can be useful regardless of the specific SDM implementation program. In the event that the program does not encompass all of the sub-categories from SDM:KOMPASS, it is possible to simply omit that sub-category. Conversely, if the program incorporates additional elements that are not present in SDM:KOMPASS, the sub-category can be included by the relevant stakeholders themselves. However, it should be noted that in many cases, departments do not even have an SDM implementation program in place. Rather, the implementation process is initiated by individuals with a strong interest in the subject matter, in which case SDM:KOMPASS will also be a valuable resource.

### The challenge of creating change–relating to the theoretical framework

Numerous studies and implementation endeavors have underscored that the integration of SDM into routine clinical practice demands a concerted effort to establish a shared comprehension among healthcare professionals regarding the purpose of patient involvement in SDM processes. This endeavor necessitates a fundamental shift in attitudes and habitual practices within the healthcare domain [1, 7, 8]. Rooted in the principles of The Normalization Process Model [16–18] and improvement methodologies [7, 8, 14, 16–18, 21–23] SDM:KOMPASS was devised to streamline the complexity of SDM implementation. Feedback from the informants in step 4 showed that the tools were deemed both understandable and useful.

A pivotal question arises: Can SDM:KOMPASS serve as a catalyst for behavioral change? In accordance with the insights of Dan and Chip Heath, it is imperative to recognize that "all change is ultimately behavior change." The Heath brothers proffer tangible methods for managing change in their seminal work “Switch–How to change things when change is hard” [35]. According to Heath we must “direct the rider” to create clarity and change. This can be done by “following the bright spots”, “script the critical moves” and “point to the destination”. In the context of SDM:KOMPASS, identifying bright spots involves selecting three to four subcategories for focused implementation, articulating the progression in each category, and elucidating the desired endpoint. SDM:KOMPASS, as a tool, can be a helpful companion in this endeavor.

Another critical aspect of effecting change is to "motivate the elephant". Heath expounds on this concept through the elements of "finding the feeling," "shrinking the change," and "growing your people." Finding the feeling, or as Heath phrases it, "creating desire," assumes paramount importance, particularly in light of studies that underscore health care professionals’ reluctance to embrace SDM for various reasons. It is well-documented that the implementation of SDM and patient decision aids is often impeded by such attitudes [11, 36]. Heath argues that change materializes when leaders effectively communicate with both the "Elephant" and the "Rider." Notably, feedback from stakeholders emphasizes SDM:KOMPASS’s value as a tool for management and leadership. Leadership is not merely confined to setting objectives and articulating a mission; it also entails fostering the desire needed to galvanize the "Elephant." Nevertheless, SDM:KOMPASS has the potential to facilitate the subsequent step of "shrinking the change." Instead of setting exceedingly ambitious goals, leaders should assist clinicians in breaking down the change into manageable components. This can be accomplished by adopting strategies such as carefully selecting sub-categories to focus on, thereby creating manageable implementation tasks—in fact, it follows a more formative approach rather than a right or wrong approach, as is often done in summative processes [37].

The third and final facet of change initiation is "Shaping the Path," which encompasses "Tweaking the Environment," "Building Habits," and "Rallying the Herd." In the context of SDM:KOMPASS, concrete guidance is provided for the environmental adjustments necessary for a meaningful implementation process. Nevertheless, many of these adjustments are context-dependent and contingent upon the specific clinical setting. The cultivation of habits partly involves training and skill development, a central theme within SDM:KOMPASS (the theme “Teaching and Qualification”, Fig 1). Notably, the leaders or facilitators of the implementation process play a pivotal role in "rallying the herd." It is well-acknowledged that the identification of a clinical champion, particularly one in a leadership position, constitutes a crucial positive factor in SDM implementation (11]. One of the categories within SDM:KOMPASS suggests the identification of clinicians possessing key competencies and entrusting them with responsibilities in the implementation process—akin to "rallying the herd."

Viewed through the lens of the insights put forth by Adam and Chip Heath, SDM:KOMPASS holds the potential to function as a facilitator of change. Although this aspect remains largely unexplored, we posit that SDM:KOMPASS will offer valuable insights into the art of directing the rider, motivating the elephant, and shaping the path of SDM implementation toward effecting sustainable change.

### Strengths and limitations

The development and initial field testing of SDM:KOMPASS present a promising advancement in SDM implementation tools and the quality improvement study should be followed by evaluations of the tool’s impact on relevant SDM implementation outcomes. Due to the nature of the tool, it may be seen and work as a recommendation of ways organizations could embed SDM into everyday practice and also help to see the maturity level of the SDM implementation process. The tool was developed with a focus on the clinical practice regarding the implementation process, and not for the purpose of developing a summative tool even though the tool’s maturity matrix format might mirrors a summative way of looking at the entire implementation process.

The strengths of the development process creating the tool relates to the solid and systematic and transparent development process which has secured solid data triangulation from experts in the field. The iterative refinement process of SDM:KOMPASS through steps 3 to 6 underscores the commitment to addressing user feedback. The inclusion of sub-categories and modifications to wording based on informant input demonstrate a responsive approach to user needs. Moreover, the extensive involvement of stakeholders in this process aligns with contemporary implementation science’s emphasis on stakeholder engagement and co-creation of tools. This practice not only enhances the tool’s alignment with end-users’ experiences but also fosters a sense of ownership and investment in its success.

### Implications for practice

Since the final workshop and user experiences in step 6 provided a promising glimpse into SDM:KOMPASS’s potential impact on real-world implementation efforts, the integration of the tool in education, planning activities, and decision-making processes signifies its usability across various contexts. The alignment between healthcare professionals’ experiences and managers’ perspectives underscores the tool’s capacity to bridge the gap between clinical and managerial realms, fostering collaboration toward a shared goal.

SDM:KOMPASS serves as a self-assessment tool tailored for healthcare professionals and managers within hospital departments, adaptable for both staff meetings and group settings, complemented by the option of external facilitation from SDM implementation consultants. The inclusivity of doctors, nurses, and pertinent staff, with no attendee limit, fosters a comprehensive approach. Each individual, regardless of prior exposure, can be equipped with the tool, while department leaders and educators are encouraged to attend SDM courses, priming them for effective SDM application.

## Conclusions

We aimed to create a practical solution for SDM implementations, acknowledging the non-linear nature of real-world settings. The result of this quality improvement study is a tool simplifying implementation steps, making the maturity of the process visible, and potentially enhancing SDM implementation’s manageability, goal-setting, and focus. While benefits are yet to be fully explored, investigations are ongoing. This study documents SDM:KOMPASS’s development, a tool supporting SDM implementation. Notably, steps 1 to 6 highlight the tool’s role in bolstering implementation, especially in complex hospital settings where managerial support is vital. The recurrent emphasis on managerial involvement underscores informants’ conscientious integration of the tool. Through an ongoing process addressing readability, usability, and alignment with real-world challenges, SDM:KOMPASS emerges as a potentially valuable resource for healthcare organizations embedding SDM. It’s structured approach, managerial effectiveness, and adaptability highlight potential for successful SDM integration. The next step will be to alpha test the tool in clinical practice.

## Supporting information

S1 Checklist Squire checklist. (PDF)

S1 Fig Guide SDM:KOMPASS. (PDF)

S1 File Interview guide no.1. Individual interviews Health care professionals.
(PDF)

S2 File Interview guide no.2. Focus group interviews Health care professionals.
(PDF)

S3 File Interview guide no.3. Individual interview with patients.
(PDF)
