# Supplementary material for: “Developing the tool SDM:KOMPASS. Supporting shared decision making implementation processes”
Source: PLoS One. 2024 Nov 18;19(11):e0312990. doi: 10.1371/journal.pone.0312990 (PMC11573207; doi:10.1371/journal.pone.0312990)
Supplement: S1 Fig — (PDF) [file pone.0312990.s002.pdf]

ABOUT SDM:KOMPASS - A TOOL SUPPORTING THE IMPLEMENTATION OF SHARED DECISION MAKING

SDM:KOMPASS aims to support the implementation of shared decision making in clinical practice.

The tool must facilitate clarification and navigation in the many facets of the implementation effort.

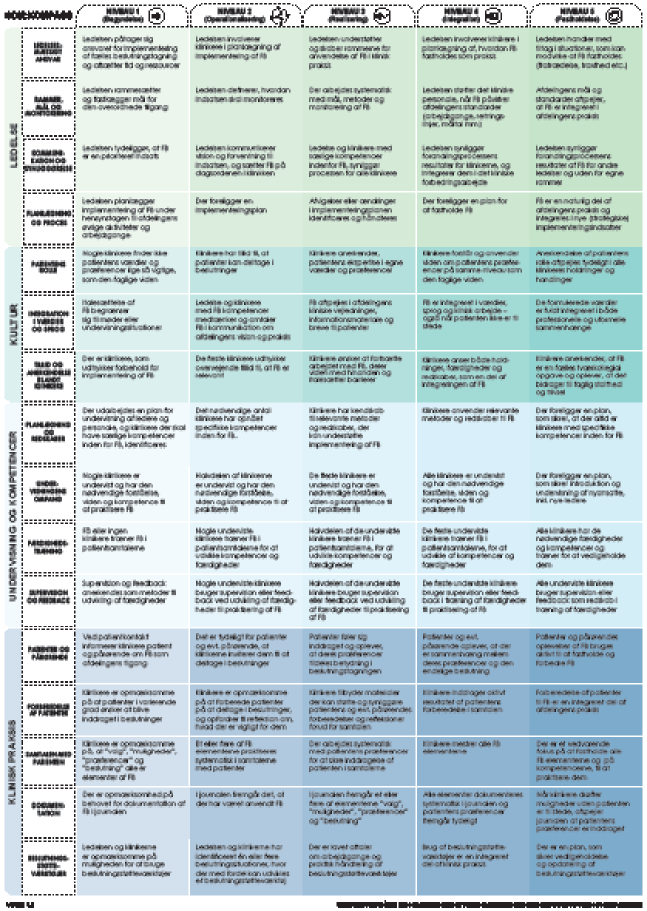

SDM:KOMPASS PROMOTES THAT:

- necessary decisions are made at management level and among the clinical staff
- local goals for the implementation effort are defined, and a strategy for implementating relevant initiatives is prepared
- the department's culture and values are further developed
- support and trust in the process as well as the new practice is ensured among managers and clinicians
- qualifications are developed, and changes in clinical practice are made
- practices are developed where shared decision making is visible to patients and relatives and in accordance with their preferences
- sustainability of shared decision making is ensured at all levels

THE STRUCTURE OF THE TOOL

SDM:KOMPASS describes relevant implementation processes within four main themes; Leadership, culture, teaching and qualifications, and clinical practices as well as related categories.

These themes and categories are described on five levels:

- **Initiation**
- **Operationalisation**
- **Implementation**
- **Integration**
- **Sustainability**

WHO CAN USE SDM:KOMPASS

SDM:KOMPASS can be used by people at management level, clinicians, consultants and teachers in shared decision making.

WHEN CAN SDM:KOMPASS BE USED

SDM:KOMPASS can be used at all times during the implementation process. The tool can serve as a guide to planning, and subsequently on how to adjust and optimize the implementation effort as well as to whether the objective has been achieved.

HOW TO USE SDM:KOMPASS

Based on the tool's structure, themes and levels, you can continuously assess where you are in the process, where you want to go, and whether you are achieving your goals. For example, you can choose to mark where you are, where you want to go, and what your next focus should be in the process of implementing shared decision making. You will often find that you are not on the same level in all categories.

SDM:KOMPASS can only be used after agreement with Center for Shared Decision Making
