# Supplementary material for: “Developing the tool SDM:KOMPASS. Supporting shared decision making implementation processes”
Source: PLoS One. 2024 Nov 18;19(11):e0312990. doi: 10.1371/journal.pone.0312990 (PMC11573207; doi:10.1371/journal.pone.0312990)
Supplement: S1 File — Individual interviews Health care professionals. (PDF) [file pone.0312990.s003.pdf]

S3 Table 3: Interview guide no.1. Individual interviews

SDM:KOMPASS - USER TEST, April-May 2021

INDIVIDUAL INTERVIEWS
Informants: Health care professionals: implementation consultants, managers, CMO, CEO

Thank you for taking the time to evaluate SDM:KOMPASS and participate in an interview.

SDM:KOMPASS aims to support the implementation of shared decision making in a clinical practice working with shared decision making. It will help illustrate and navigate the many facets of implementation efforts.

As an important part of the development process, we are interested in hearing from key persons about their experiences with the tool. We are very grateful that you will participate.

SDM:KOMPASS can be used at any stage of an implementation process. In the planning phase, the tool can serve as a guide for planning implementation, and then for adjusting and optimizing implementation efforts and goal achievement.

My interview with you consists of four parts:
1. You read the tool and think aloud at the same time
2. You receive a short questionnaire that you fill out
3. I ask you about your thoughts and reactions as you read through the SDM:KOMPASS tool
4. I have some additional questions I would like to ask you

If it's OK with you, I'd like to record my interview with you - then I can focus entirely on listening and asking questions.

Upon consent, receive consent

Is there anything you would like to ask before we begin?

S3 Table 3: Interview guide no.1. Individual interviews

Part I:
Imagine that you need to use the tool as a guide in your department's work to implement shared decision-making. What level are you at, and where do you want to be? Consider whether the tool will help you improve your efforts to implement shared decision-making. Feel free to point out what level your department is at. Think out loud as you read and evaluate. I am listening, but we are not going to talk yet.
Interviewer; Remember to time the interview

Part II:
Fill out the questionnaire and give it to the interviewer.

Part III:
Based on the interviewer's observations: ask for thoughts and reactions while reviewing the SDM:KOMPASS tool.

Part IV:
Semi-structured interview with interview guide

1. Try to describe the department or purpose of the assessment that you were thinking about when you went through SDM:KOMPASS
2. Try to describe how you did it.
    a. How did you read the tool?
    b. What levels fit your department? Do they match your immediate impression of the department's efforts and status?
3. What is your immediate assessment of SDM:KOMPASS?
4. Are the headings of the four themes and categories understandable (self-explanatory)?
5. Do the four themes include what you think is relevant to implementation? (exhaustive)
    a. Have we included the necessary aspects?
6. Do you think Level 5 describes a department that has successfully implemented shared decision-making?
7. What do you think about departments at Level 1?
8. If you read horizontally from left to right: Is there a logical progression in the level of implementation for each category?
9. Do you think SDM:KOMPASS can be used to describe the implementation process of different departments?
10. Can the SDM:KOMPASS help motivate clinicians and managers and guide the implementation process going forward?
11. Do you think managers and clinicians (doctors, nurses, teach-the-teachers) will assess the level of a department in the same way?
12. Is there anything else
    a. distracting overlap or missing aspects?
    b. can you imagine using SDM:KOMPASS - and possibly recommending it to others?

Thank you for your time!
