# Supplementary material for: “Developing the tool SDM:KOMPASS. Supporting shared decision making implementation processes”
Source: PLoS One. 2024 Nov 18;19(11):e0312990. doi: 10.1371/journal.pone.0312990 (PMC11573207; doi:10.1371/journal.pone.0312990)
Supplement: S2 File — Focus group interviews Health care professionals. (PDF) [file pone.0312990.s004.pdf]

S4 Table 4: Interview guide no.2. Focus group interviews

SDM:KOMPASS - USER TEST 2, April-May 2021

GROUP INTERVIEWS
Informants: Health care professionals: implementation consultants

SDM:KOMPASS and the guide for SDM:KOMPASS are read in advance.

Thank you for taking the time to evaluate SDM:KOMPASS and participate in an interview. SDM:KOMPASS aims to support the implementation of shared decision-making in a clinical practice working with shared decision-making. It will help illustrate and navigate the many facets of implementation efforts. As an important part of the development process, we are interested in hearing from key persons about their experiences with the tool. We are very grateful that you will participate. SDM:KOMPASS can be used at any stage of an implementation process. In the planning phase, the tool can serve as a guide for planning implementation, and then for adjusting and optimizing implementation efforts and goal achievement.

1: Collect questionnaire and consent
2: Focus group interview - semi-structured interview with interview guide

If it's OK with you, I'd like to record your contribution - then I can focus entirely on listening and asking questions. Is there anything you would like to ask before we begin?

1. What is your immediate assessment of SDM:KOMPASS?
2. Are the headings of the four themes and categories understandable (self-explanatory)?
3. Do the four themes include what you think is relevant to implementation? (exhaustive)
    a. Have we included the necessary aspects?
4. Do you think Level 5 describes a department that has successfully implemented shared decision-making?
5. What do you think about departments at Level 1?
6. If you read horizontally from left to right: Is there a logical progression in the level of implementation for each category?
7. Do you think that SDM:KOMPASS can be used to describe the implementation process of different departments?
8. Can the SDM:KOMPASS help motivate clinicians and managers and guide the implementation process going forward?
9. Do you think managers and clinicians (doctors, nurses, teach-the-teachers) will assess the level of a department in the same way?
10. Is there anything else
    a. distracting overlap or missing aspects?
    b. the instructions?
    c. how long it will take to read and assess a department?
    d. can you imagine using the SDM:KOMPASS - and possibly recommending it to others?

Thank you for your time!
