# Supplementary material for: “Developing the tool SDM:KOMPASS. Supporting shared decision making implementation processes”
Source: PLoS One. 2024 Nov 18;19(11):e0312990. doi: 10.1371/journal.pone.0312990 (PMC11573207; doi:10.1371/journal.pone.0312990)
Supplement: S3 File — Individual interview with patients. (PDF) [file pone.0312990.s005.pdf]

S5 Table 5: Interview guide no.3. Individual interviews with patients

---

SDM:KOMPASS - USER TEST, April-May 2021

INDIVIDUAL INTERVIEWS
Informants: Patients

All three sub-categories; The Patient’s role , Patients and Relatives, and Preparation of Patients are included in the interview.

1. Explanation of the tool in general with purpose, target group (This is especially important because it is also an explanation of why they should not test the entire tool), themes, categories, levels.
2. Ask them to read through the sub-categories to test and ask them to think out loud. Make notes (just like with solo interviews - so we keep the stringency)
3. What are your immediate thoughts?
4. What do you think about the way we have described the patient's role in Shared Decision Making?
5. When reading horizontally from left to right: Is there a logical progression in the gradation for each category? (question from the interview guide)
6. Are we ambitious enough?
7. Is there anything you think is missing or something that should be removed?
8. Any other comments?
